# Integrated 3D printing and case-based learning in orthopedic residency education for geriatric hip fracture management

**DOI:** 10.3389/fsurg.2025.1659207

**Published:** 2025-09-26

**Authors:** Honglin Wang, Tao Yang, Wei Hua, Wentao Zhang, Lin Lu

**Affiliations:** 1 Department of Orthopedics, Renmin Hospital of Wuhan University, Wuhan, China; 2 Department of Hepatobiliary Surgery, Renmin Hospital of Wuhan University, Wuhan, Hubei, China

**Keywords:** medical education, case-based learning, 3D printing, standardized training of residents, geriatric hip fracture

## Abstract

**Objective:**

Geriatric hip fractures, clinically designated as “the last fracture in life” due to significant disability and mortality, pose critical educational barriers for orthopedic residents in mastering complex hip anatomy and surgical decision-making competencies. This study evaluates the implementation value of integrating digital 3D-printing technology with CBL pedagogy in standardized residency training for geriatric hip fracture management.

**Methods:**

Fifty-six orthopedic residents undergoing standardized training at Renmin Hospital of Wuhan University were enrolled and randomly assigned to either the control group (CBL, *n* = 28) or the experimental group (CBL-3DP, *n* = 28). Both groups received training in surgical planning for geriatric hip fractures. The CBL group underwent traditional CBL teaching, while the CBL-3DP group combined CBL with 3D-printed fracture models. Post-training assessments evaluated theoretical knowledge, practical skills, satisfaction, and engagement with the teaching methodology.

**Results:**

Following the instructional intervention, the CBL-3DP group demonstrated significantly superior performance compared to the conventional CBL group in both theoretical and practical assessments. Theoretically, the CBL-3DP cohort achieved higher scores in regional anatomy comprehension, fracture classification accuracy, and mastery of treatment principles (*p* < 0.05). Practically, this group exhibited enhanced competencies in geriatric hip fracture management domains including patient consultation, physical examination, diagnostic precision, basic emergency management, and preoperative surgical planning (*p* < 0.05). Questionnaire analyses further indicated that the 3D + CBL approach yielded significantly better outcomes than traditional CBL in: depth of understanding of geriatric hip fractures, learning enthusiasm, diagnostic capabilities, surgical planning proficiency, confidence in managing clinical cases, and satisfaction with the instructional methodology.

**Conclusion:**

The integration of 3D printing with CBL methodology enhances training effectiveness and learner satisfaction in geriatric hip fracture education, supporting its adoption in standardized orthopedic residency programs.

## Introduction

Hip fracture, a prevalent traumatic injury among elderly osteoporotic patients, is often termed the “last fracture in life” due to its associated high rates of disability and mortality (1, 2). Mastery of standardized diagnosis and treatment protocols for this condition constitutes a core component of orthopedic residency training, requiring trainees to acquire proficiency in complex surgical planning and comprehensive patient assessment within constrained rotation periods (3). However, traditional teaching methods frequently yield suboptimal outcomes. Given the intricate anatomy of the hip joint combined with the frequent comorbidity of osteoporosis in elderly patients, fracture patterns exhibit considerable variability, surgical options become diverse yet technically demanding, and the imperative for holistic evaluation of underlying comorbidities creates significant challenges (4). Consequently, achieving comprehensive competency proves difficult for trainees. Furthermore, the prevalent reliance on didactic, instructor-centered teaching approaches among some educators further diminishes pedagogical effectiveness (5).

In recent years, Case-Based Learning (CBL) has gained prominence in clinical education owing to its practical, heuristic, and targeted nature (6, 7). This student-centered approach fosters clinical reasoning and problem-solving skills through the analysis of authentic cases (8). Within geriatric hip fracture education, CBL incorporating plain radiographs and three-dimensional computed tomography (3D-CT) enhances trainees’ theoretical understanding. Nevertheless, for residents with limited surgical expertise and clinical exposure, reliance solely on 2D/3D imaging impedes the three-dimensional conceptualization of fracture mechanisms, precise classification, assessment of displacement severity, and consequently, the selection of optimal surgical strategies and fixation devices/prostheses (9). Concurrently, existing synthetic bone models inadequately simulate the tactile experience and complexities of reducing and fixing intricate fractures (10). Consequently, there is a critical need for visualization tools capable of accurately and intuitively displaying fracture morphology while facilitating preoperative simulation, thereby overcoming current pedagogical limitations.

Three-dimensional (3D) printing technology, which fabricates physical models through the layer-by-layer deposition of materials based on digital designs, has attracted significant interest in medicine since its emergence (11). This technology enables the rapid and convenient generation of three-dimensional pathological anatomical models. In orthopedics, it has demonstrated substantial value for creating patient-specific instruments, enhancing preoperative planning, improving intraoperative guidance, and augmenting patient comprehension of surgical procedures (12–14). Studies indicate that 3D-printed models significantly enhance surgical residency education (15). The 3D-printed models preserved core fracture characteristics (displacement, comminution, and continuity), essential for achieving surgical planning realism. Compared to static image review, junior surgeons and medical students achieve a more intuitive grasp of complex anatomy and can practice procedural simulations on the models (10). This model-based pedagogical approach facilitates a paradigm shift from passive knowledge transmission to active inquiry-based learning. It proves instrumental in stimulating engagement and promoting knowledge transfer (16).

Presently, the efficacy of integrating 3D-printed models with CBL for residency training in geriatric hip fracture management remains undetermined. This study aims to evaluate the utility of personalized 3D-printed models combined with the CBL methodology in this specific clinical training context, thereby providing novel approaches and a foundational reference for advancing instructional quality.

## Materials and methods

### Participant characteristics

Fifty-six residency trainees (2022–2023 cohort) with no prior orthopedic rotation experience were enrolled. Inclusion criteria: (1) informed consent; (2) proficient communication/comprehension skills; (3) completion of all teaching tasks; (4) completion of assessments/satisfaction surveys. Exclusion criteria: (1) absenteeism; (2) non-compliance with teaching; (3) severe communication barriers. Participants retained the right to withdraw without justification.

Using computer-generated randomization, trainees were allocated to either the CBL group (*n* = 28) or CBL-3DP group (*n* = 28). Baseline characteristics (gender, age, education level, years post-graduation) showed no intergroup differences.

### 3D printed model generation

Typical geriatric hip fracture cases (femoral neck, intertrochanteric, subtrochanteric fractures with comorbidities) were selected via Renmin Hospital's PACS. Imaging data (x-ray, CT scans with reconstructions) were collected with informed consent to establish a fracture classification database.

**Figure 1:**
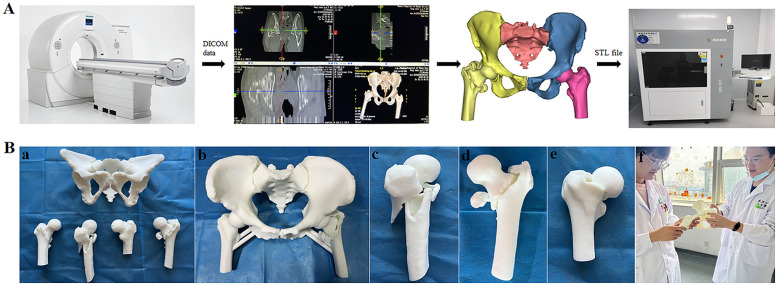
**(A)** Flow chart of Hip fracture model printing. **(B)** 3D printed model of geriatric hip fracture patients. Figure legends: **(a)** Overall view of 3D-printed models from three geriatric hip fracture patients. **(b)** 3D-printed model of an intertrochanteric fracture patient. **(c)** 3D-printed femoral model of a subtrochanteric fracture patient. **(d)** 3D-printed femoral model of an intertrochanteric fracture patient. **(e)** 3D-printed femoral model of a femoral neck fracture patient. **(f)** In-class teaching session.

Model fidelity is a critical factor for surgical planning. To achieve this, DICOM files from raw CT scans (slice thickness ≤0.625 mm, 64-slice CT) were reconstructed into 3D fracture models using Mimics 19.0 software (Materialise, Belgium). During segmentation, all fracture fragments exceeding 1 mm in size were manually annotated through layered color masking, while smaller bone fragments underwent micro-peg docking design prior to export as independent STL files with customized supports. These models were printed at 1:1 scale via stereolithography (SLA) technology (UnionTech Lite 600 2.0) using medical-grade photopolymer resin, with critical fidelity-preserving measures including a 0.1-mm layer thickness, UV-resistant surface encapsulation after post-curing to prevent fracture line erosion during repeated handling, and dissolvable PVA-supported micro-peg assemblies for fragment fixation (Figure 1A). The resultant models encompassed complex intertrochanteric, femoral neck, and subtrochanteric fracture types for standardized surgical training (Figure 1B). The 3D-printing process required an average duration of 5 hours per model, with a unit cost of CNY 1,000.

### Teaching methodology

Both cohorts received standardized instruction from the same three board-certified orthopedic surgeons (mean experience: 8.2 ± 1.3 years) following identical syllabi and schedules. The core curriculum encompassed four domains: (1) Regional anatomy, (2) Fracture classification, (3) Perioperative management protocols, and (4) Evidence-based surgical planning. Mandatory asynchronous video-based learning on hip anatomy and fracture surgery was completed prior to formal instruction, with pre-session examinations assessing resident trainees’ readiness for video-based learning. Trainees subsequently completed the training in rotating cohorts through a 4-week program consisting of three 2-hour didactic sessions weekly.

Within the CBL group, trainees formed 4-member learning cohorts receiving tiered instruction from the same faculty. Supervising physicians curated standardized teaching modules featuring complete clinical cases (medical histories, diagnostic records, imaging data, surgical notes) to facilitate systematic analysis. Sessions progressively explored surgical indications/contraindications, fracture classification, displacement patterns, and preoperative planning through peer-driven discussion. Each case culminated in a trainee-led surgical plan presentation, with faculty providing comprehensive feedback post-discussion. During case analyses, participants could pause discussions to seek peer or faculty clarification before proceeding.

In contrast, the CBL-3DP group integrated physical 3D-printed fracture models into the CBL methodology. Beyond standard case discussions, instructors utilized tactile models to demonstrate fracture principles, anatomical relationships, classification systems, and corresponding treatment plan. Clinical decision-making exercises incorporated active manipulation of 3D models, followed by hands-on surgical pathway planning and simulated procedures on these models. Following initial simulations, faculty conducted secondary model-based reviews to reinforce fracture mechanics comprehension and optimize surgical strategies through iterative practice.

Both groups concluded with summative faculty commentary addressing common controversies and performing competency assessments via 3D model simulations.

### Evaluating teaching effectiveness

Following the completion of the training program, post-instructional assessments were conducted using institution-developed theoretical examinations and practical competency evaluation scales. These instruments comprehensively evaluated knowledge domains including pathoanatomy, clinical manifestations, diagnosis, and management of geriatric hip fractures. To maintain assessor blinding and mitigate bias, personnel involved in teaching were excluded from assessment, all evaluation materials were anonymized, and a standardized assessment protocol was administered by an independent coordinator.

The theoretical examination (100-point scale) comprised multiple-choice questions, short-answer items, and case analyses assessing hip anatomy, fracture classification, treatment principles, and surgical techniques. The practical assessment (100-point scale) evaluated medical history taking, clinical documentation, radiographic interpretation, perioperative management, and preoperative surgical planning, with higher scores indicating superior performance.

Additionally, a validated 6-item structured questionnaire employing a 5-point Likert scale (1 = strongly disagree to 5 = strongly agree) was administered to assess subjective learning experiences. This instrument measured: depth of understanding regarding geriatric hip fractures, learning enthusiasm, diagnostic capabilities, surgical planning proficiency, confidence in clinical case management, and satisfaction with the instructional methodology. The internal consistency of the questionnaire was assessed by calculating Cronbach's alpha using IBM SPSS Statistics (Version 27.0.1). The obtained value was 0.712, which is slightly above the minimum acceptable threshold of 0.70.

### Statistical analysis

Data analysis was performed using SPSS 22.0. Continuous variables were expressed as mean ± standard deviation (SD), with intergroup comparisons analyzed via independent samples *t*-tests and intragroup comparisons via paired *t*-tests. Categorical variables were assessed using *χ*² tests. Statistical significance was established at *p* > 0.05.

## Results

### Characteristics of enrolled residents

As detailed in Table 1, fifty-six surgical residents completed the study protocol. The CBL group comprised 25 males and 3 females (age range: 23–33 years; mean age: 27.47 ± 2.65 years), while the CBL-3DP group included 26 males and 2 females (age range: 25–34 years; mean age: 28.26 ± 2.54 years). No statistically significant differences (*p* > 0.05) were observed between groups regarding educational background, gender distribution, age, or years post-graduation.

**Table 1:** Comparison of the baseline characteristics.

Item	Educational attainment (bachelor's/master's and above)	Sex(female/male)	Age(years)	Years since undergraduate graduation (years)
CBL group (*n* = 28)	5/23	3/25	27.470 ± 2.650	4.200 ± 2.000
CBL-3DP group (*n* = 28)	4/24	2/26	28.260 ± 2.540	4.800 ± 1.800
*χ*²/t value	0.134	0.220	−1.139	−1.137
*p* value	0.714	0.639	0.260	0.261

### Theoretical and practical performance

No significant differences were observed in pre-instruction assessment scores between the two groups. Post-training evaluations revealed superior performance in the CBL-3DP cohort across all metrics. The CBL-3DP group demonstrated significantly higher theoretical scores (71.14 ± 3.95 vs. 62.53 ± 4.24; t = 7.861, *p* < 0.001), practical assessment scores (64.54 ± 4.96 vs. 54.93 ± 3.11; t = 8.677, *p* < 0.001), and composite scores (135.68 ± 8.89 vs. 117.46 ± 6.61; t = 8.696, *p* < 0.001) compared to the CBL group (Table 2).

**Table 2:** Intergroup assessment scores: CBL and CBL-3DP groups.

Item	CBL group	CBL-3DP group	t value	*p* value
Total pre-course score	105.25 ± 5.73	111.50 ± 8.43	0.904	0.370
Pre-course Theoretical knowledge score	54.46 ± 3.05	55.29 ± 3.08	1.071	0.289
Pre-course Practical skill score	50.79 ± 2.69	51.29 ± 3.08	0.725	0.471
Total post- course score	117.46 ± 6.61	135.68 ± 8.89	8.696	<0.001
Post-course Theoretical knowledge score	62.53 ± 4.24	71.14 ± 3.95	7.861	<0.001
Post-course Practical skill score	54.93 ± 3.11	64.54 ± 4.96	8.677	<0.001

**Figure 2:**
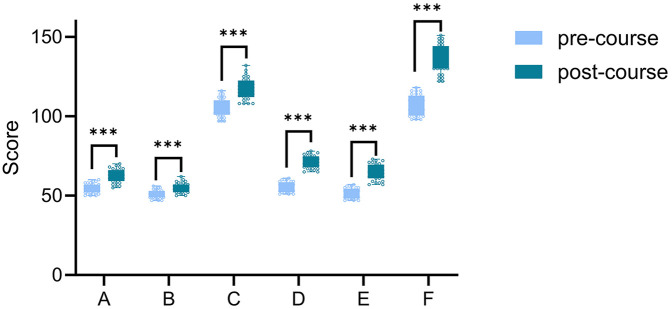
Intragroup score comparison: Pre- vs. Post-course. Figure legends: A&D, B&E and C&F show comparisons of the theoretical, practical, and total scores of the CBL group and the CBL + 3D group before and after class, respectively. *** indicates *p* < 0.001.

Furthermore, both groups exhibited statistically significant improvements in theoretical and composite scores when comparing pre- and post-course assessments (Figure 2).

### Questionnaire results

**Figure 3:**
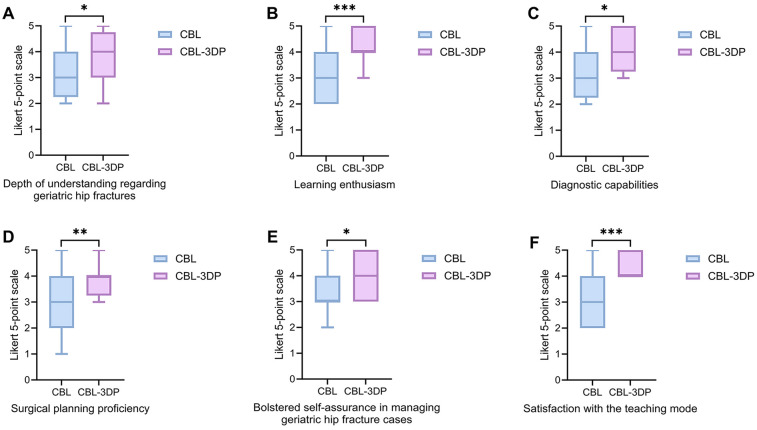
Five-point Likert scores of residents’ attitudes in CBL and CBL-3DP groups. **(A)** Depth of understanding regarding geriatric hip fractures. **(B)** Learning enthusiasm. **(C)** Diagnostic capabilities. **(D)** Surgical planning proficiency. **(E)** Bolstered self-assurance in managing geriatric hip fracture cases. **(F)** Satisfaction with the teaching mode, *p < 0.05, **p < 0.01, ***p < 0.001.

All 56 participants completed and returned the questionnaires. Comparative analysis revealed that the CBL-3DP group demonstrated statistically significant improvements across multiple dimensions relative to the CBL group. These enhancements encompassed: depth of understanding regarding geriatric hip fractures, learning enthusiasm, diagnostic capabilities, surgical planning proficiency, confidence in managing geriatric hip fracture cases, and satisfaction with the teaching mode (Table 3 and Figure 3). All improvements reached statistical significance (*p* < 0.05).

**Table 3:** Comparison of questionnaire results between the two groups.

Item	CBL	CBL-3DP	t value	*p* value
Depth of understanding regarding geriatric hip fractures	3.143 ± 0.848	3.679 ± 0.983	2.183	0.033
Learning enthusiasm	3.214 ± 1.101	4.143 ± 0.651	3.843	<0.001
Diagnostic capabilities	3.429 ± 1.103	4.071 ± 0.766	2.532	0.015
Surgical planning proficiency	2.929 ± 1.412	3.857 ± 0.591	3.209	0.003
Bolstered self-assurance in managing geriatric hip fracture cases	3.357 ± 0.951	3.964 ± 0.838	2.534	0.014
Satisfaction with the teaching mode	3.321 ± 1.090	4.321 ± 0.476	4.448	<0.001

## Discussion

Residency standardized training represents a critical pathway for enhancing clinical competencies, where evidence-based pedagogical approaches are essential for advancing diagnostic and therapeutic proficiency (17). Orthopedics—a core surgical subspecialty characterized by its broad disease spectrum, intricate anatomical and biomechanical dependencies, and reliance on three-dimensional spatial cognition —demands exceptional practical skills (14, 18, 19). High-quality orthopedic training thus establishes a fundamental foundation for surgical practice. Geriatric hip fractures, as high-risk injuries in osteoporotic populations, constitute a central training objective due to their prevalence, frequent comorbidities, and elevated morbidity/mortality under conservative management (e.g., hypostatic pneumonia, pressure ulcers, deep vein thrombosis) (20). Surgical intervention remains the gold standard, requiring physicians to master complex 3D hip anatomy and spatial reasoning (21).

Conventional teaching methods relying on anatomical atlases, imaging data (x-ray/CT/MRI), and 2D reconstructions inadequately convey the spatial complexity of hip anatomy, fracture pattern variability, or comorbidity management logic (22). These limitations hinder trainees’ imaging interpretation, spatial cognition, and clinical decision-making, necessitating pedagogical innovation (23).

CBL provides an effective pathway to overcome traditional limitations (7). Using authentic clinical cases as educational vehicles, CBL employs a closed-loop cycle of case exposure to self-directed inquiry to faculty guidance. This cycle drives trainees’ active integration of core knowledge, including anatomical structures, imaging characteristics, and therapeutic principles (24). In hip fracture education, this methodology facilitates collaborative group discussions focused on clinical problems, enabling systematic analysis of fracture mechanisms, surgical planning, and complication prevention strategies. This process progressively builds a comprehensive clinical reasoning pathway from imaging interpretation to treatment decisions. Unlike traditional apprenticeship models—which often foster theory-practice disconnects, inadequate clinical examination skills, and poor comprehension of surgical logic through instructor-dominated teaching (3) —CBL centers on student-led problem identification, analysis, and resolution. This approach not ensures thorough mastery of syllabus-mandated theoretical knowledge (8), but crucially develops trainees’ innovative thinking, clinical history-taking proficiency, diagnostic synthesis capabilities, and evidence-based decision-making competencies (25). Consequently, CBL significantly elevates both educational quality and trainees’ professional competence.

At the surgical planning stage, 2D imaging constraints and generic models’ non-specificity introduce deeper cognitive barriers. 3D printing overcomes this by generating patient-specific bone models that physically manifest fracture line trajectories, fragment displacement vectors, and articular surface relationships—transforming abstract anatomy into tactile reality (26). Our study integrates 3D-printed models into CBL through faculty-guided collaborative sessions where trainees observe pathology morphology and spatial relationships on these models while designing and simulating surgical plans such as implant positioning, reduction pathways, and cup/intramedullary nail placement. This process enables crucial validation of reduction feasibility and implant fit, translating theoretical principles into concrete preoperative rehearsals. This hands-on verification transcends technical training—it elucidates biomechanical relationships between fracture patterns and fixation strategies, allowing trainees to anticipate surgical challenges during planning and significantly boost decision confidence (13). Naturally integrated within CBL case discussions, this process establishes a closed-loop pathway of cognitive construction (CBL) to tactile verification (3D -printed models) to plan optimization, effectively transforming passive learning into active exploration.

The dual-track pedagogical model—integrating theoretical training (CBL clinical reasoning development) with tangible practice (3D-printed decision validation)—demonstrated significant advantages. Our findings indicate that trainees using this approach achieved substantially higher scores than the CBL-only control group in both theoretical assessments and clinical operative skills evaluations (*p* < 0.05), confirming the model's feasibility and superiority. Self-evaluation questionnaires also revealed significantly improved teaching satisfaction (*p* < 0.05), indicating trainees’ strong preference for this deeply integrated visual-practical methodology. This integrated approach also enhanced mastery of hip fracture management principles and functional reconstruction processes. Compared to conventional CBL, 3D model-enhanced CBL enabled comprehensive spatial visualization of neurovascular anatomy and three-dimensional relationships around the hip (27). Furthermore, 3D printing technology generated customized models (varying scales or specific cross-sections) from single imaging datasets according to diverse teaching needs (16). Unconstrained by geographical limitations, this approach improved instructional efficiency, optimized pedagogical workflows, and accelerated the learning curve (15).

Beyond geriatric hip fractures, 3D-printed models demonstrate broad educational utility across orthopedic subspecialties (23, 28, 29). Applications include multi-angle tumor boundary visualization for preoperative planning (28), fracture classification accuracy exceeding radiographic assessment (23), and time-efficient implant templating (29), collectively substantiating 3D printing's universal educational value. It should be noted that the cost of 3D-printed models varies significantly, typically ranging from hundreds to thousands of US dollars, depending on model complexity, materials, equipment, and software. The total cost (including materials and processing) for the hip fracture model used in this study was approximately CNY 1,000, representing a relatively economical option for current educational training. As 3D printing technology and software continue to advance, associated costs are expected to decrease steadily, thereby enhancing feasibility for implementation in resident training programs. Furthermore, extended reality (XR) technologies—encompassing Virtual Reality (VR), Augmented Reality (AR), and Mixed Reality (MR)—serve as valuable teaching tools in orthopedics and related fields (9, 30, 31). Their application in scenarios such as fracture pattern training effectively addresses the limitations of high-cost physical 3D models and CT imaging, offering practical alternatives or complementary solutions for cost-sensitive settings.

More significantly, our integrated 3DP-CBL pedagogy converges with pioneering medical education research (27, 32). In developmental dysplasia of the hip (DDH) training, Feng et al. (27) demonstrated 3DP-CBL's superiority over traditional CBL, significantly enhancing theoretical knowledge acquisition, clinical skills proficiency, learner engagement, and satisfaction. Similarly, Zhao's team demonstrated enhanced content mastery, advanced critical thinking, and refined clinical reasoning through 3DP-CBL implementation in tetralogy of Fallot instruction (32). Collectively, these findings substantiate that synergistic integration of 3D printing and CBL constructs an extensible methodological architecture for medical education, propelling standardized innovation in educational practice.

This study has several limitations. The small sample size (*n* = 56) and single-institution recruitment may introduce selection bias and limit the generalizability of the findings. Additionally, the non-blinded design along with post-randomization consent procedures could lead to further selection bias. Although the self-designed questionnaire showed acceptable internal consistency (Cronbach's α > 0.7), it lacks formal validation (such as factor analysis), which may affect the robustness of subjective outcome measures. Furthermore, the study assessed only short-term teaching effects, with no evaluation of long-term knowledge retention or skill transfer. Therefore, to enhance the validity and applicability of future findings, large-scale, multi-center trials are recommended. Further validation of the assessment tool through cognitive interviews or factor analysis would strengthen its construct validity. Long-term follow-up studies are also needed to evaluate the sustained educational impact of this teaching model. Expanding research to other orthopedic areas, such as knee and spinal disorders, could further demonstrate its broader utility.

In conclusion, the integration of 3D printing with CBL methodology enhances educational outcomes and learner satisfaction in geriatric hip fracture training, meriting its implementation and broader adoption in standardized orthopedic residency programs.

## Data Availability

The raw data supporting the conclusions of this article will be made available by the authors, without undue reservation.
